# Emotional dyscontrol in multiple sclerosis: an opinion article

**DOI:** 10.3389/fnbeh.2024.1376021

**Published:** 2024-04-10

**Authors:** Mara Palumbo, Sara Palumbo

**Affiliations:** Department of Clinical and Experimental Medicine, University of Pisa, Pisa, Italy

**Keywords:** emotional dyscontrol, social cognition, emotion recognition, multiple sclerosis, theory of mind

## Highlights:

- Emotional dyscontrol is often underdiagnosed in patients with MS. The lack of consensus regarding classification and diagnostic criteria necessitates a more detailed definition of emotional dyscontrol and standardized diagnostic questionnaires.
- Emotional dyscontrol is frequently underreported. The presence of stigma surrounding emotional dyscontrol often results in individuals refraining from reporting it to parents and clinicians. Increasing awareness in clinical settings by incorporating the assessment of emotional dyscontrol within the clinical evaluation of this condition is necessary.
- Knowledge about the specific domains of social cognition affected in MS is still limited. Personalized assessment for specific social cognitive deficits using standardized tasks for evaluating different domains of theory of mind or emotion recognition is recommended.
- Knowledge about the links between deficits in different domains of social cognition, specific neurological damage, neurochemical changes, and emotional dysregulation is limited. A personalized characterization of these aspects in relation to each other is suggested at the patient level.
- Deficits in specific social cognitive domains, including emotion recognition and theory of mind, are thought to underscore limited social interactions and emotional dyscontrol, which impact social and psychological quality of life of individuals with multiple sclerosis. Recognizing and addressing these issues may enhance the general well-being of individuals with MS.

## Introduction

The overall quality of life and wellbeing of individuals with multiple sclerosis (MS) are dramatically affected by cognitive deficits (present in 40%−70% of patients), and impairments in the domains of social cognition further contribute to affecting their lives (see Cotter et al., 2016 for a systematic review and meta-analysis). Social cognition deficits encompass challenges in emotion recognition and theory of mind, which, in turn, are also directly linked to the emotional dyscontrol observed in certain MS patients (Cotter et al., 2016).

Emotional dyscontrol refers to difficulties in regulating emotions underlying episodes of inappropriate or exaggerated emotional responses, including uncontrollable laughing, crying, or anger that appear to be disproportionate to the context (Arciniegas and Wortzel, 2018).

Emotional dyscontrol is common in mental disorders, including schizophrenia, bipolar disorder, borderline personality disorder (Sharma and McClellan, 2021), as well as in traumatic and non-traumatic brain injuries (Annoni et al., 2006; Bryant et al., 2022), and neurological disorders, including MS (Wortzel et al., 2008; Finegan et al., 2019). For example, a study observed that 73% of the participants with MS reported symptoms of emotional dyscontrol in the month preceding enrollment (Feinstein and Feinstein, 2001). Moreover, 8%−46% of patients with MS also receive a diagnosis of pathological laughter and crying-a disorder of affect characterizing several neurological disorders, characterized by difficulty controlling emotional expressions of laugher and crying, exaggerated in response to the actual emotional valence of contextual information- usually in later stages of the disease (Feinstein et al., 1997; Parvizi et al., 2006; Hanna et al., 2016; Luhoway et al., 2019). Importantly, it has been pointed out that even though they clearly have a profound negative impact on the patient's wellbeing, emotional dysfunctions are somehow still underreported to clinicians, underdiagnosed, and undertreated (Luhoway et al., 2019). Among the possible reasons of these problems, stigma surrounding these conditions, inconsistency among definitions, and a lack of standardized assessment tools have been suggested (Luhoway et al., 2019).

In individuals with MS, emotional dyscontrol often manifests as a profound and foolish apathy (called “emotional blunting”) both toward others and various aspects of everyday life, boosts of laughter that appear senseless and lack any apparent cause, and episodes of irritability, sadness, and tearfulness without apparent reasons (Feinstein and Feinstein, 2001).

In MS, emotional dyscontrol is believed to act as a mediator for the effects of the disease on neuropsychiatric disturbances (Prakash et al., 2019), thereby indicating it as a potential intermediate phenotype. Indeed, MS patients have an increased lifetime risk for neuropsychiatric disturbances, including major depression, and symptoms of depression and anxiety, which often manifest in comorbidity with emotional dyscontrol (Prakash et al., 2019; Schirda et al., 2020). Even though some patients with MS do not fully meet the criteria for the diagnosis of a mental disorder, the coexistence of emotional dyscontrol with depressive and/or anxiety symptoms has been shown to significantly affect the degree of psychological stress and the quality of life of MS patients (Feinstein and Feinstein, 2001).

This evidence highlights the importance of deepening scientific knowledge about emotional dyscontrol in patients with MS, emphasizing the need to further research in this area with the aim of developing targeted interventions to address these problems and improve the quality of life for patients with MS.

## Exploring the relationship between multiple sclerosis and emotional dyscontrol

MS is a chronic inflammatory, demyelinating, and neurodegenerative disease that affects the central nervous system of young adults (Filippi et al., 2018). It is characterized by damage to nerve fibers and axonal pathology, as well as microstructural changes and brain atrophy, resulting in a diverse range of symptoms (Feinstein et al., 1997; Hanna et al., 2016). These symptoms can affect various parts of the body and cause both physical and cognitive impairments of varying severity, depending on the brain region involved and the degree of demyelination (Zagon and McLaughlin, 2017; Filippi et al., 2018).

The exact etiological mechanisms underlying emotional dyscontrol in MS ere not fully understood because, to date, this aspect has been poorly explored. One of the main obstacles to research in this area is that, in addition to the psychiatric manifestations of MS, many patients develop psychological problems in reaction to the disease (Haussleiter et al., 2009). Reactive psychological problems have been challenging to distinguish and separate from the psychopathology directly related to the morphological changes that occur in the brains of MS patients (Haussleiter et al., 2009).

Nevertheless, because brain demyelination and inflammation processes typical of MS can affect neural pathways involved in emotional regulation, they might also underlie the emotional dyscontrol observed in patients (Krause et al., 2009). The structural and functional integrity within the ventral paralimbic network, responsible for emotion regulation, the dorsal paralimbic network, facilitating conscious perception and intentional emotional control, and the cerebellum, which is interconnected with and regulates both networks, may be compromised in neurological disorders, including MS; these mechanisms have been hypothesized to underlie emotional dyscontrol (Wortzel et al., 2008).

Furthermore, in MS patients, emotional dyscontrol has been shown to significantly correlate with specific cognitive indices of executive functions, such as, for example, Stroop test, divided attention, and verbal fluency (Feinstein et al., 1999; Berneiser et al., 2014; Pfaff et al., 2021). As these indices rely on frontal lobe functioning, this evidence linking executive dysfunctions with emotional dyscontrol is in line with the hypothesis of a prefrontal involvement in the mechanisms underlying emotional dyscontrol in MS patients (Feinstein et al., 1999).

## Theory of mind and facial emotion recognition in MS patients

A recent meta-analysis has revealed significant impairments in overall theory of mind abilities, and its cognitive and affective components in all subtypes of MS, including primary-progressive, secondary-progressive, and relapsing-remitting MS (Lin et al., 2021). The same meta-analysis also revealed impairments in emotion recognition, which were not exhibited homogeneously across patients with different subtypes of MS (Lin et al., 2021).

Indeed, in MS, emotional dyscontrol has been shown to be associated with difficulties in recognizing facial expressions, linked to the use of inappropriate eye-gaze strategies to decode facial emotions (Henry et al., 2009, 2011; Krause et al., 2009; Banati et al., 2010; Berneiser et al., 2014). One study, investigating a cohort of 52 patients with different types of MS, found that 21% of them had developed significant facial emotion recognition impairments (Polet et al., 2023). These deficits appeared to be particularly pronounced for negative emotions, mainly fear and anger (Henry et al., 2009, 2011; Berneiser et al., 2014; Pfaff et al., 2021), but also sadness (Krause et al., 2009; Berneiser et al., 2014; Pfaff et al., 2021) and disgust (Lin et al., 2021). Deficits in recognition of surprise and neutral faces were also reported (Lin et al., 2021; Pfaff et al., 2021).

The heightened impairment of patients with MS in experiencing fear and anger has been hypothesized to be related with abnormalities in the structure and function of bilateral and right amygdala (Green et al., 2018; Pitteri et al., 2019).

Moreover, it has been hypothesized that emotion recognition deficits in MS patients might result also from temporal white matter lesions, which could impair the transmission of information from temporal visual processing areas, important for facial processing, to frontal regulation areas, such as the ventro-lateral prefrontal cortex that selectively activates when observing the eye region and controls facial emotion recognition (Krause et al., 2009).

The difficulties in emotional dimensions do not appear to be influenced by the severity of physical disability or the duration of the disease (Pfaff et al., 2021). However, facial emotion recognition deficits have been shown to be more frequent and severe in progressive forms (Polet et al., 2023). In line with this evidence, a recent study has compared emotion recognition performance of patients with relapsing-remitting MS with that of individuals affected by secondary-progressive MS, suggesting that emotion recognition dysfunctions might be a sign of MS progression (Argento et al., 2022). Additionally, the authors observed that emotion recognition deficits were linked to poorer cognitive performance (i.e., verbal learning and memory, abstract reasoning, executive function efficiency, and overall cognitive impairment) only in relapsing-remitting MS patients (Argento et al., 2022). On the opposite, in the secondary-progressive MS patients, emotion recognition deficits strongly correlated with psychological aspects, such as perceived fatigue, anxiety, depression, and anger but not with cognitive performance (Argento et al., 2022). In particular, only in these subgroup of MS patients, high levels of anger (trait, state, and expression), lowered the ability to decode other's emotion during the Reading the Mind in the Eyes test.

Overall, it has been hypothesized that patients with the secondary-progressive form might experience more emotional dysregulations than those with relapsing-remitting MS, as they develop difficulties both in managing their own emotions and in recognizing the emotions of others (Argento et al., 2022). Interestingly, fatigue and impairment in social cognition have been linked to reduced function in the right prefrontal and anterior cingulate cortex, as well as damage to frontal white matter fibers (Neuhaus et al., 2018). Similar neurological alterations might characterize patients with secondary-progressive MS experiencing emotional dyscontrol.

The most severe emotional dysregulations appear to affect severely physically disabled patients, probably due to more extensive brain damage, coupled with more severe cognitive impairments, particularly in verbal fluency, verbal learning, and auditory recall (Feinstein et al., 1997; Hanna et al., 2016). These severely ill patients are more prone to exhibiting pathological laughter and crying.

The brain lesions causing the latter condition are very heterogenous; dysfunctions in a cortico-limbic-subcortico-thalamo-ponto-cerebellar network have been hypothesized, where symptoms mirror both direct effects of lesions occurring in crucial brain arears and indirect effects where these areas are not lesioned but are influenced by functionally connected lesioned areas (a mechanisms termed diaschisis) (Klingbeil et al., 2021). A bilateral network has been proposed, where a loss of control by cortical areas on two affected neural systems, one involved in emotional regulation and the other involved in volitional control, takes place (Klingbeil et al., 2021).

The above scientific data suggest that emotional dyscontrol in MS patients follows different paths in the context of psychological and/or neurological conditions and is influenced by the type and anatomical localization of brain lesions.

## Discussion

Emotional dyscontrol significantly affects the overall quality of life of patients affected by MS (Phillips et al., 2014). It has been proposed that emotional control abilities should be investigated in MS to consider potential targeted interventions for correcting emotional dyscontrol, if present, with the ultimate goal of enhancing the patient's quality of life (Phillips et al., 2014). Other authors have highlighted that interventions should be considered also when such disabilities do not reach the severity necessary to be considered pathological, that is, fulfilling a psychiatric diagnosis (Feinstein and Feinstein, 2001).

Indeed, emotional dysfunctions might be linked to social functioning impairments negatively effecting overall wellbeing. The diminished ability of MS patients with emotional dysfunctions to interpret facial expressions could, in part, underlie alterations of their social interaction abilities. In fact, the ability to identify emotional expressions allows exhibiting appropriate behavioral responses and is pivotal for proper social interaction (Muratori et al., 2024). However, knowledge about the specific domains of social cognition affected in MS is still limited (Neuhaus et al., 2018). A better characterization of the social cognition deficits underpinning emotional dyscontrol and their neural correlates is necessary to understand whether different paths of dyscontrol stem from deficits in different domains of social cognition associated with different neurological deficits and/or patterns of brain lesion. This understanding is crucial not only for distinguishing emotional dyscontrol between different subtypes of MS, but also between patients form the same subgroup with diverse clinical symptoms.

Overall, the scientific literature concerning the relation between MS, emotional dyscontrol, and quality of life of patients suggests that emotion recognition should receive more attentions in clinical setting, be incorporated within the clinical evaluation of this disease, and treated.

Tailored interventions aimed at improving deficits in social cognition skills, such as, for example, emotion recognition training or mindfulness meditation, might be considered as potential treatments for reducing emotion dysregulation and improve social functioning and quality of life for patients affected by MS (Schirda et al., 2020). For example, a computerized social cognition training has been shown effective in improving recognition of facial emotions (Schoeneman Patel et al., 2022). At last, pharmacological treatments have been shown to be effective for treating pathological laughing and crying (Wortzel et al., 2008; Husbands and Talbot, 2022) and might be beneficial also for treating emotional dyscontrol in MS.

## Author contributions

MP: Writing – original draft. SP: Writing – original draft, Writing – review & editing.
